# Food cravings in food addiction: exploring a potential cut-off value of the Food Cravings Questionnaire-Trait-reduced

**DOI:** 10.1007/s40519-017-0452-3

**Published:** 2017-10-28

**Authors:** Adrian Meule

**Affiliations:** 1 Department of Psychology, University of Salzburg, Hellbrunner Straße 34, 5020 Salzburg, Austria; 2 Center for Cognitive Neuroscience, University of Salzburg, Salzburg, Austria

**Keywords:** Food craving, Food addiction, Food Cravings Questionnaire-Trait, Yale Food Addiction Scale

## Abstract

**Purpose:**

The Food Cravings Questionnaires are among the most often used measures for assessing the frequency and intensity of food craving experiences. However, there is a lack of studies that have examined specific cut-off scores that may indicate pathologically elevated levels of food cravings.

**Methods:**

Receiver-Operating-Characteristic analysis was used to determine sensitivity and specificity of scores on the Food Cravings Questionnaire-Trait-reduced (FCQ-T-r) for discriminating between individuals with (*n* = 43) and without (*n* = 389) “food addiction” as assessed with the Yale Food Addiction Scale 2.0.

**Results:**

A cut-off score of 50 on the FCQ-T-r discriminated between individuals with and without “food addiction” with high sensitivity (85%) and specificity (93%).

**Conclusions:**

FCQ-T-r scores of 50 and higher may indicate clinically relevant levels of trait food craving.

**Level of evidence:**

Level V, descriptive study.

## Introduction

Food craving is an intense desire to eat a specific food [1, 2]. The Food Cravings Questionnaires (FCQs [3, 4]) are two of the most widely used measures for the assessment of food cravings. While the 15-item state version (FCQ-S) measures the intensity of food craving in the current moment, the 39-item trait version (FCQ-T) measures the frequency and intensity of food craving experiences in general. Experiencing a food craving often precipitates binge-eating and, accordingly, higher FCQ-T scores are associated with higher eating disorder pathology, such as more frequent binge-eating episodes [5, 6]. Similarly, individuals with bulimia nervosa and binge-eating disorder report higher FCQ-T scores than healthy controls and FCQ-T scores can discriminate between anorexia and bulimia nervosa subtypes [7–10].

Recently, a 15-item short form of the FCQ-T—the FCQ-T-reduced (FCQ-T-r)—has been proposed [11]. While the scale was initially developed in German, its one-factorial structure and correlates have since been replicated with a Spanish [12], Italian [13, 14], and English version [15]. The FCQ-T-r has high internal consistency and test–retest-reliability [16]. Furthermore, FCQ-T-r scores predicted food cravings in daily life, thus supporting ecological validity of the scale [17].

Since publication of the FCQ-T-r, I have often received requests about a potential cut-off value, higher scores of which may indicate clinically relevant levels of “trait food craving”. While momentarily experiencing a food craving is common and does not indicate pathological eating behavior per se, the above-mentioned findings suggest that elevated trait food craving scores may indeed do so. Therefore, the current report aims to provide researchers and practitioners with a potential cut-off score on the FCQ-T-r that might be used for evaluating whether an individual’s food craving experiences are so frequent and intense that they might be considered pathologically elevated.

Craving experiences are a pivotal characteristic of substance use disorders [18]. By translating diagnostic criteria of substance use disorder to food and eating, researchers have aimed at assessing addiction-like eating behavior, which is usually operationalized with the Yale Food Addiction Scale (YFAS, which has been recently revised to a YFAS 2.0 [19]). Studies showed that classifications of “food addiction” according to the YFAS strongly overlap with the diagnoses of established eating disorders, such as anorexia nervosa binge–purge subtype, bulimia nervosa, and binge-eating disorder [20–23]. As FCQ-T scores have been found to be elevated in these binge-eating-related disorders as well as in those with “food addiction” [24], the dichotomous YFAS score (“food addiction” vs. “no food addiction”) may be well-suited as a transdiagnostic criterion for establishing a cut-off value of the FCQ-T-r. Therefore, data from a previously reported study, in which both the FCQ-T-r and the YFAS 2.0 were used [25], were re-analyzed to explore a potential cut-off score of the FCQ-T-r that would differentiate between individuals with and without “food addiction”.

## Methods

### Participants

Participants represented a convenience sample that completed an online questionnaire, which included the FCQ-T-r and the YFAS 2.0. Recruitment procedure and other details are reported elsewhere [25]. For the current analyses, data of *n* = 432 participants were available (88.4% female, *n* = 382). Most participants were students (78.9%, *n* = 341) and had German citizenship (82.9%, *n* = 358). Mean age was 25.6 years (*SD* = 7.09, Range: 16–55) and mean BMI was 22.3 kg/m^2^ (*SD* = 3.70, range 12.2–42.5). Most participants had normal weight (77.5%, *n* = 335, BMI = 18.5–24.9 kg/m^2^) and few were underweight (6.90%, *n* = 30, BMI < 18.5 kg/m^2^), overweight (11.6%, *n* = 50, BMI = 25.0-29.9 kg/m^2^), or obese (3.90%, *n* = 17, BMI ≥ 30.0 kg/m^2^).

### Measures

*Food Cravings Questionnaire-Trait-reduced (FCQ-T-r)*. The 15-item FCQ-T-r measures the frequency and intensity of food craving experiences in general [11]. Items are scored on a six-point scale from *never*/*not applicable* (1) to *always* (6). Thus, sum scores can range between 15 and 90 with higher scores indicating more frequent and intense food cravings. In the current study, mean FCQ-T-r score was 34.5 (*SD* = 14.5, range 15–84) and internal consistency was *α* = 0.953.

*Yale Food Addiction Scale* (*YFAS*) *2.0*. The 35-item YFAS 2.0 measures addiction-like eating behavior based on the eleven diagnostic criteria for substance use disorder in the fifth revision of the Diagnostic and Statistical Manual of Mental Disorders [19]. Items are scored on an eight-point scale from *never* (0) to *every day* (7). All items are then dichotomized, whereby there are different cut-off values for the single items. A symptom count can be calculated by adding up all endorsed symptoms and, thus, this score can range between 0 and 11 symptoms. Moreover, a dichotomous score (“food addiction” vs. no “food addiction”) can be calculated. A classification as “food addicted” is indicated by meeting at least two symptoms and, additionally, requires the presence of clinically significant impairment or distress. In the current study, mean number of endorsed “food addiction” symptoms was 1.32 (*SD* = 2.52, range 0–11) and 43 participants (10.0%) were classified as “food addicted”. Internal consistency was *α* = 0.947.

### Data analyses

According to Bühner [26], one approach to find a criterion for differentiating between a non-clinical and a clinical population is to consider test scores of two standard deviations below the mean of the clinical population and two standard deviations above the mean of the non-clinical population. This criterion was used as a first approximation to a potential cut-off value of the FCQ-T-r. As a next step, a Receiver-Operating-Characteristic (ROC) analysis was calculated with IBM SPSS Statistics Version 20 to determine sensitivity and specificity of FCQ-T-r scores for differentiating between individuals with and without “food addiction”.

## Results

**Fig. 1:**
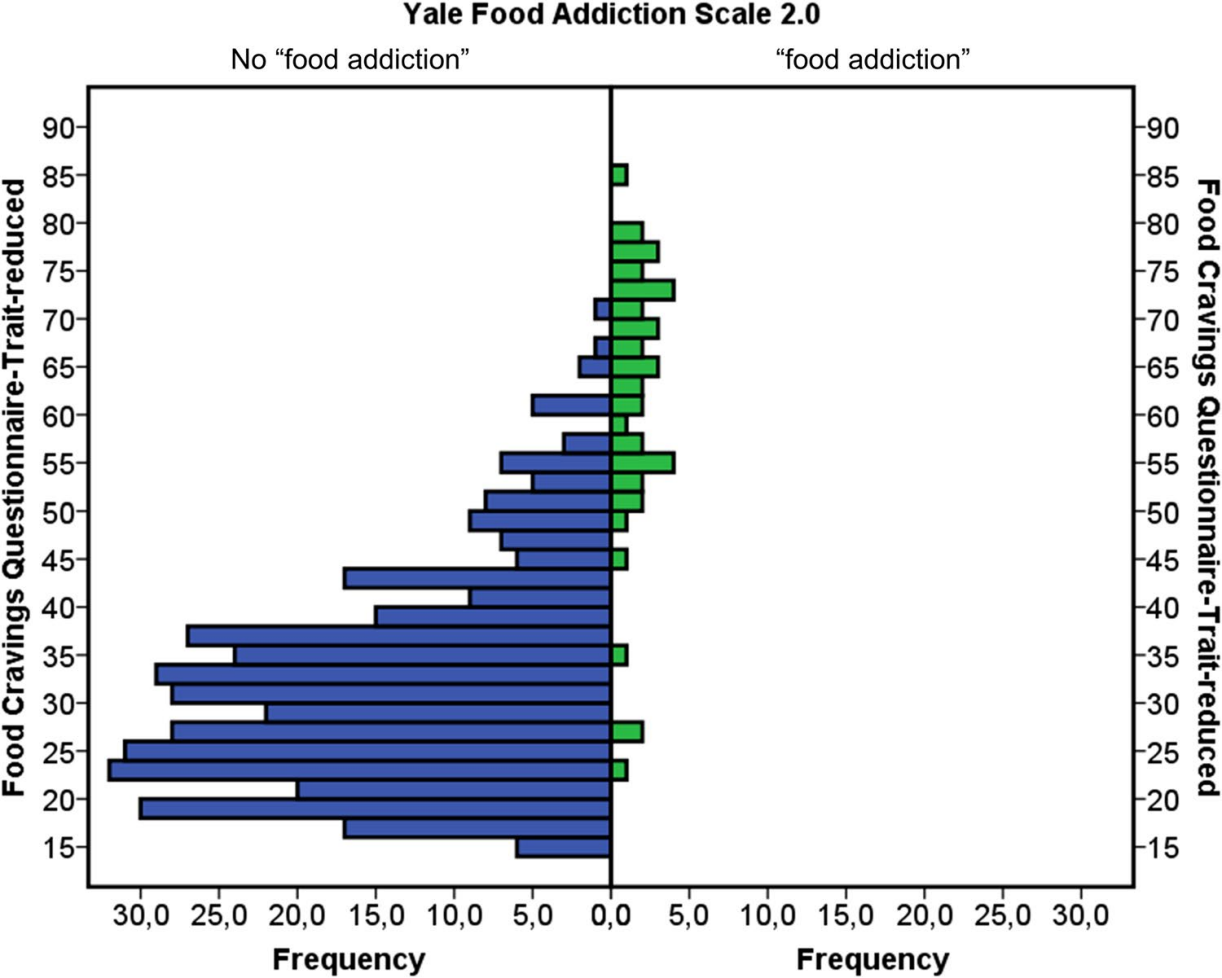
Frequencies of scores on the Food Cravings Questionnaire-Trait-reduced as a function of group (no “food addiction” vs. “food addiction”) as classified with the Yale Food Addiction Scale 2.0

**Fig. 2:**
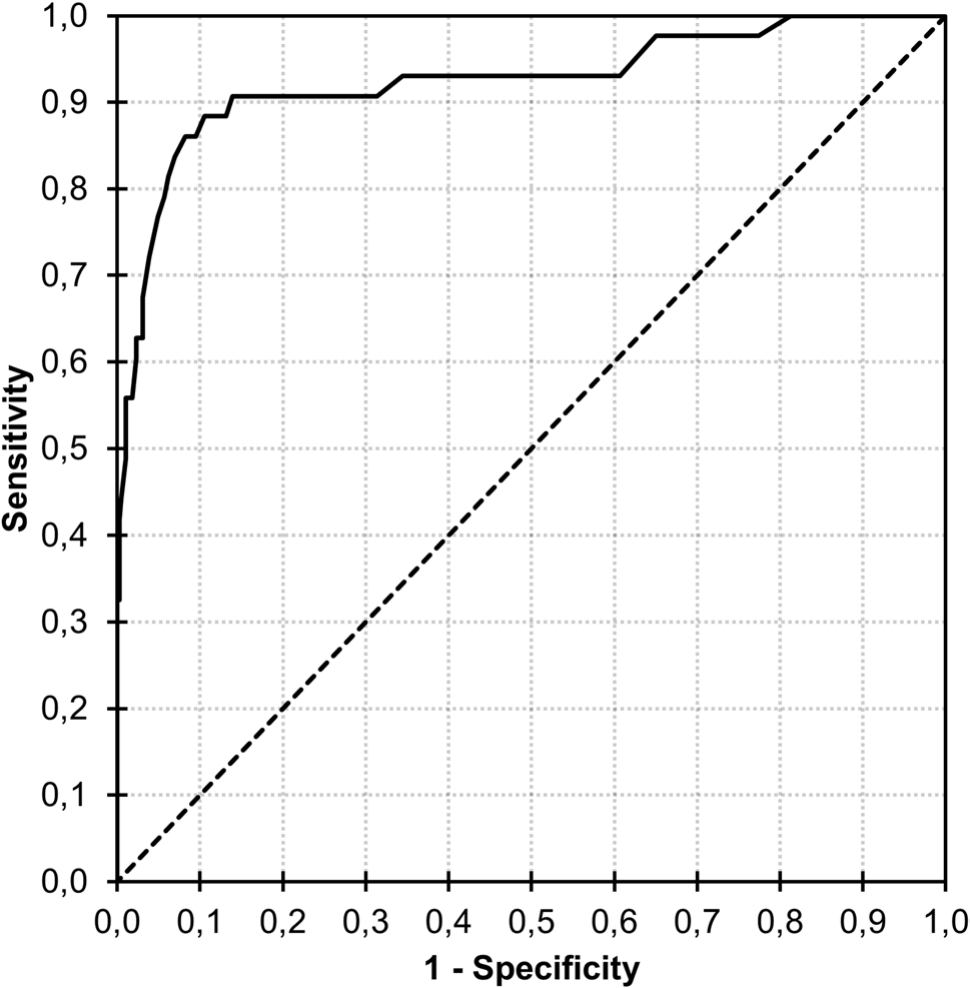
Receiver-Operating-Characteristic curve for discriminating between individuals with and without “food addiction” by means of scores on the Food Cravings Questionnaire-Trait-reduced

Frequencies of FCQ-T-r scores as a function of group are displayed in Fig. 1. Mean FCQ-T-r scores were 31.5 (*SD* = 11.1) in the group without “food addiction” and 61.0 (*SD* = 14.6) in the group with “food addiction” [*t*_(430)_ = 16.0, *p* < 0.001, *d* = 2.57, 95% CI (2.21–2.93)]. Thus, using the criterion of ±2 *SD* would suggest a range for a potential cut-off value between 61.0 − (2 × 14.6) = 31.8 and 31.5 + (2 × 11.1) = 53.7. In the ROC analysis, area under the curve was 0.925 [*SE* = 0.03, *p* < 0.001, 95% CI (0.872, 0.979); Fig. 2]. Sensitivity and specificity values were reasonably high (> 80%) at FCQ-T-r scores between 41 and 52 (Table 1). For example, sensitivity was 84.9% and specificity was 92.5% at an FCQ-T-r score of 50.

**Table 1:** Sensitivity and specificity at different scores on the Food Cravings Questionnaire-Trait-reduced (FCQ-T-r) for discriminating between individuals with and without “food addiction”

FCQ-T-r score	Sensitivity	Specificity
32	0.930	0.568
33	0.930	0.605
34	0.930	0.640
35	0.919	0.671
36	0.907	0.709
37	0.907	0.744
38	0.907	0.767
39	0.907	0.786
40	0.907	0.799
41	0.907	0.811
42	0.907	0.834
43	0.907	0.856
44	0.896	0.865
45	0.884	0.873
46	0.884	0.880
47	0.884	0.889
48	0.872	0.900
49	0.860	0.911
50	0.849	0.925
51	0.826	0.935
52	0.803	0.941
53	0.779	0.947
54	0.744	0.956

## Discussion

Results suggested a range between 32 and 54 for a potential cut-off value of FCQ-T-r scores that differentiate between the absence and presence of “food addiction”. Using a cut-off score of 50 on the FCQ-T-r, high sensitivity and specificity for discriminating individuals with and without “food addiction” were demonstrated. Notably, this score is equal to one standard deviation (*SD* = 15) above the mean FCQ-T-r score (*M* = 35) in the current study. This resonates well with previous studies that examined high and low trait food craving at ±1 *SD* from the samples’ mean FCQ-T-r score and in which high scores (i.e., one standard deviation above the samples’ mean) approximated a value of 50 [e.g., 17, 27].

In contrast, Innamorati and colleagues [13] found that a score of 57.5 on the Italian FCQ-T-r differentiated between different binge-eating severities. However, this score achieved rather unsatisfactory specificity and sensitivity (69% each). Furthermore, this score may overestimate an appropriate cut-off value as analysis was based on the discrimination between moderate vs. severe binge-eating levels (thus, excluding individuals without binge-eating symptomatology).

In conclusion, the current study provides preliminary evidence for a potential cut-off score of the FCQ-T-r that may indicate clinically relevant levels of trait food craving. However, analysis was based on a self-selected sample and a self-report outcome measure. Therefore, future studies need to examine the usefulness of the cut-off value proposed in the current report for differentiating between more representative healthy samples and clinical samples with established eating disorder diagnoses, such as bulimia nervosa or binge-eating disorder.
